# The Birth Prevalence of Mucopolysaccharidosis Types I, II, III, IVA, VI, and VII in the Republic of Kazakhstan Between 1984 and 2023

**DOI:** 10.3390/diagnostics15060679

**Published:** 2025-03-10

**Authors:** Assel Tulebayeva, Gulnar Mukhambetova, Maira Sharipova, Anna Tylki-Szymanska

**Affiliations:** 1 Paediatric Department, Kazakh National Medical University, Almaty 050012, Kazakhstan; muhambetova.g@kaznmu.kz; 2 Scientific Center of the Pediatrics and Pediatric Surgery JSC, Almaty 050060, Kazakhstan; m.sharipova@pediatria.kz; 3 The Children’s Memorial Health Institute, 04-730 Warsaw, Poland; atylki@op.pl

**Keywords:** birth prevalence, mucopolysaccharidoses, the Republic of Kazakhstan

## Abstract

**Objectives:** Mucopolysaccharidoses (MPSs) are a group of a rare inherited lysosomal storage diseases caused by a deficiency or complete lack of lysosomal enzymes participating in glycosaminoglycan (GAG) degradation, which leads to multisystemic impairment and early mortality. This study aimed to determine the birth prevalence of MPS type I, II, III, IVA, VI, and VII in the Republic of Kazakhstan. **Methods:** Retrospective epidemiological calculations were carried out on all enzymatically and genetically confirmed MPS cases diagnosed between 1984 and 2023 in the Republic of Kazakhstan. Birth prevalence was calculated by dividing the number of patients diagnosed with MPS by the total number of live births in the same period, recalculated for every 100,000 live births. **Results:** The overall birth prevalence of MPS was 0.77 per 100,000 live births. The highest birth prevalence was MPS II with 0.36 (47% of all diagnosed MPS types), followed by MPS I with 0.16 (21%), MPS VI with 0.12 (16%), MPS IVA with 0.09 (11%), MPS IIIB with 0.03 (4%), and MPS VII (which is the rarest type) with 0.007 (1%). **Conclusions:** The most common MPS type in the Republic of Kazakhstan is MPS II (Hunter syndrome).

## 1. Introduction

Mucopolysaccharidoses (MPSs) are a group of rare genetic diseases caused by defects of lysosomal enzymes participating in glycosaminoglycan (GAG) degradation. The active accumulation of non-degraded GAGs in all connective tissue structures in MPS leads to multisystemic impairment and is subsequent to multiorgan dysfunction. At present, there are eight different types of MPSs (I, II, III, IV, VI, VII, IX, and X) known to be caused by 12 insufficient lysosomal enzymes [1,2]. All MPS types are inherited in an autosomal recessive manner, except MPS II (Hunter syndrome), which is transmitted in an X-linked recessive manner [3].

The total MPS incidence rate varies from 1.35 to 16.9 per 100,000 live births in different countries (Table 1). The smallest MPS incidence rate among inhabitants of European countries is fixed in Sweden and Denmark (1.75 and 1.77, respectively), while it accounted for 4.5 per 100,000 live births in the Netherlands [4,5,6]. The lowest and highest global MPS incidence rates, 1.35 and 16.9 per 100,000 live births, have been reported in South Korea and Saudi Arabia, respectively, with the latter being due to the high rate of marriages among first cousins (around 40%) and relatives (up to 60%) [7,8,9]. Among all MPS types, MPS III prevails in Europe and Australia [10], while this type is rare in Saudi Arabia, Northern Ireland, and China, where it accounts for 11%, 9%, and 3.7%, respectively [11,12,13]. MPS II is predominantly diagnosed in East Asian countries, such as South Korea, Taiwan, China, Japan, and Malaysia, comprising 54.6%, 52%, 47.2%, 53%, and 39.6% of all MPS types, respectively [9,11,12,14,15]. MPS II is also frequently diagnosed among the Ashkenazi Jewish population, as well as in Portugal, Brazil, and Estonia, accounting for 34%, 37%, and 53% of all MPS types, respectively [16,17,18,19]. However, MPS II is rare in Australia and Norway, accounting for 9% and 4% of all MPS types, respectively; moreover, there are no diagnosed cases of MPS II in Saudi Arabia [6,7,10]. According to the literature, MPS I is the most common MPS type in Canada, Denmark, Norway, Sweden, and Northern Ireland, accounting for 30%, 30%, 60%, 38%, and 41% of all MPS types, respectively [6,13], but is the rarest MPS type in Taiwan, at only 6% [14]. MPS IVA (Morquio syndrome) is rare in Australia, with a birth prevalence of 0.16 per 100,000 live births, and accounts for 4.5% of all types of MPS; similarly, Brazil, Portugal, and the Netherlands also report a low number of cases, comprising 11%, 10%, and 6% of all MPS types, respectively. There are no confirmed cases of MPS IV in Estonia [18]. MPS VI (Maroteaux–Lamy syndrome) is the most-diagnosed MPS type in Saudi Arabia (with a birth prevalence of 7.85 per 100,000 live births) and India, accounting for 46% and 26% of all MPS types, respectively [7,20,21]. However, MPS VI is rarely diagnosed in South Korea, Norway, Sweden, Poland, Estonia, and Germany, comprising 1.4%, 2%, 4%, 1%, 7%, and 7% of all MPS types, respectively; there are no data regarding patients with MPS VI in Northern Ireland [4,6,9,13,18,22]. MPS VII is an ultra-rare type among all MPSs, as there are no diagnosed cases of MPS VII in many countries. According to the literature review, the highest birth prevalence of MPS VII among all other MPS types is in Mexico at 0.23 per 100,000 live births; whereas in Australia, Czech Republic, Japan, the United States, and Switzerland, MPS VII is the rarest type and accounted for 0.047, 0.02, 0.02, 0.027, and 0.038, respectively. Thus, MPS incidence rates in European and Asian countries vary considerably. There is no information regarding the birth prevalence of MPSs in Central Asian countries. Epidemiological studies on MPSs have not been conducted in the Republic of Kazakhstan; as a result, the birth prevalence of MPSs in the Republic of Kazakhstan is unknown. The birth prevalence of all MPS types in different countries is presented in Table 1.

## 2. Materials and Methods

This study included patients diagnosed with MPS who were receiving follow-up treatment in the Scientific Center for Pediatrics and Pediatric Surgery and the National Scientific Center for Maternity and Childhood from 1984 to 2023. In Kazakhstan, all MPS cases are registered in these two main pediatric centers. MPS diagnosis was based on the enzyme activity assay and molecular genetic testing in dried blood spot cards (DBSs). The diagnosis based on molecular genetic testing started in Kazakhstan in 2015; before 2015, all patients with MPS were diagnosed only based on the enzyme activity assay. Due to the lack of specialized laboratories in the Republic of Kazakhstan, these studies were carried out in the laboratories of the University Medical Center Hamburg-Eppendorf and the Centogene laboratory (Germany), the Laboratory of Archimed Life Science GmbH (Austria), and the Laboratory of Molecular Genetics and Cell Biology of the Scientific Center for Children’s Health (Russian Federation). The assessment of lysosomal enzyme activity was measured in DBSs by fluorometry (based on the fluorometric determination of 4-methylumbelliferone), using high-performance liquid chromatography and tandem mass spectrometry. The *IDUA*, *IDS*, *NAGLU*, *GALNS*, *ARSB*, and *GUSB* genes were analyzed by PCR and the sequencing of the entire coding region and the highly conserved exon–intron splice junctions. The assessment of lysosomal enzyme activity and genetic testing in DBSs comprise the screening method for several lysosomal storage diseases (LSDs, including Gaucher disease, Fabry disease, MPS, GM1 gangliosidosis, Niemann–Pick A/B disease, and Tay–Sachs and Sandhoff diseases) due to minimal invasiveness and easy collection, storage, and transport. According to the international workshop sponsored by Genzyme Corporation in 2009 (Amsterdam, The Netherlands) that had 40 expert participants in LSD diagnostics and screening programs from Europe, the USA, South America, and Asia, DBS testing proved to be a quick and practical method for LSD diagnoses [27].

To calculate the MPS birth prevalence, statistical data on the annual birth rate of children in the Republic of Kazakhstan were used [28]. Birth prevalence was calculated by dividing the number of patients diagnosed with MPS by the total number of live births in the period between the birth of the eldest and youngest patients, recalculated per 100,000 live births [5,12,23,24].

## 3. Results

MPS is a rare disease in Kazakhstan, and its birth prevalence is challenging to estimate. The program for diagnosing MPS in children and adolescents, by determining lysosomal enzyme activity in leukocytes and carrying out molecular genetic testing in clinically and phenotypically suspected patients, was launched for the first time in the Republic of Kazakhstan in 2009.

During the study period (1984–2023), different types of MPS were diagnosed in 105 children and adolescents in the Republic of Kazakhstan as follows: MPS I—22 (21%); MPS II—49 (47%); MPS IIIB—4 (4%); MPS IVA—12 (11%); MPS VI—17 (16%); and MPS VII—1 (1%). All diagnosed patients were under observation in the Scientific Center of Pediatrics and Pediatric Surgery and National Scientific Center for Maternity and Childhood. Among the MPS patients, some were siblings: two children each in three families with MPS I; two children in six families with MPS II; two siblings in two families with MPS IIIB; three children in one family and two siblings in another with MPS IVA; and two siblings in three families with MPS VI. Four patients were born from consanguineous marriages: two siblings with MPS I, one child with MPS I, and one girl with MPS VI.

The birth prevalence calculation was carried out for the period of 1984 to 2023, where the number of MPS patients was divided by the total number of live births for the same period, as was the period when MPS patients were born, and expressed per 100,000 live births.

The retrospective analysis was conducted for 105 patients diagnosed with MPS I, II, IIIB, IVA, VI, and VII. Between 1984 and 2023, 13,666,750 children were born in the Republic of Kazakhstan. The overall MPS birth prevalence was 0.77 per 100,000 live births. The birth prevalence of all types is listed as follows, as shown in Table 2: MPS I—0.16; MPS II—0.36; MPS IIIB—0.03; MPS IVA—0.09; MPS VI—0.12; and MPS VII—0.007 per 100,000 live births. However, these numbers do not represent the actual birth prevalence of MPS, since only children clinically and phenotypically suspected of having MPS were diagnosed.

Given the data presented above, MPS II can be considered the most common type in the Republic of Kazakhstan. Table 3 provides information about the groups of patients with MPS I, II, IIIB, IVA, VI, and VII in the Republic of Kazakhstan.

During the study period, among the 105 MPS patients, there were 13 cases of mortality (12.4%) associated with cardiopulmonary insufficiency due to respiratory infection: 2 siblings with MPS I died at the ages of 5 and 6 years; 6 boys with MPS II at the ages of 5, 7, 10, 11, 15, and 24 years; 1 patient with MPS IIIB at the age of 19; 1 patient with MPS IVA, due to severe atlantoaxial instability, at the age of 7 years; 2 patients with MPS VI at the ages of 5 and 27 years; and 1 patient with MPS VI, due to a tragic car accident. All patients were on enzyme replacement therapy (ERT) for an average of 2.8 years (1–4 years), except for 1 patient who passed away at the age of 24 years with MPS II and another patient with MPS IIIB. The reasons for ERT absence were as follows: for MPS II, ERT was not available or registered in Kazakhstan in 2006; for MPS IIIB, treatment had not been developed yet. All aforementioned patients had severe progressive forms of the disease: Hurler syndrome; a neuronopathic form of Hunter syndrome; a severe form of MPS IIIB; progressive atlantoaxial instability in a patient with MPS IVA; severe narrowed craniovertebral junction in a patient with MPS VI, who died in a car accident.

## 4. Discussion

Our results on the birth prevalence of MPS are similar to data from Asian countries, where the most frequent is MPS II (Hunter syndrome), with a birth prevalence of 0.36 per 100,000, accounting for 47% of all MPS types. This is higher than the data in European countries, such as Norway, Denmark, and Sweden (Table 1), but much lower than the results of epidemiological studies conducted in Asian countries. For instance, Taiwan, Japan, and South Korea had MPS II birth prevalences of 1.07, 0.84, and 0.74 per 100,000 live births (notably, 2.05 per 100,000 live births of boys in Taiwan), accounting for 52%, >50%, and 54.6% of all diagnosed MPS cases, respectively. One reason for the high MPS II incidence rates could be the inheritance pattern of MPS II, which is X-linked recessive. Thus, in families where two or more sisters are carriers of the pathogenic X chromosome, the probability of having male children with MPS II disease is high.

The second most common type of MPS in Kazakhstan is MPS I (21%), with a birth prevalence of 0.16 per 100,000 live births, which is three times lower than in the Czech Republic (0.72) and Sweden (0.67), but higher than in Taiwan (6%), South Korea (15.3%), and Estonia, where no cases of MPS I were diagnosed. According to our data, the two most common mutations were c.1709A>T (p.Asp570Val) and c.208C>T (p.Gln70Ter), the latter mainly diagnosed in patients of Russian nationality. E. Yu Voskoboeva et al. have reported that c.208C>T (p.Gln70Ter) is the most prevalent mutation among Russian and Tatar patients, which could be explained by the founder effect [29].

MPS VI is the third most common in the Republic of Kazakhstan, with a birth prevalence of 0.12 per 100,000 live births (16% among all MPS types) and much higher than several European countries, including Estonia (7%), Germany (7%), Norway (2%), Sweden (4%), and Poland (1%).

MPS IVA showed a low birth prevalence of 0.09 per 100,000 live births (11%) in Kazakhstan, which is significantly lower than European countries (Sweden—0.38; Denmark—0.48; Norway—0.76; Germany—0.38; and Northern Ireland—1.3) and Asian countries (Taiwan—0.33; South Korea—0.25; and Japan—0.15). MPS IVA is not prevalent in any country in the world and, thus, considered rare; it comprised 4.5%, 11%, 6%, and 10% of all MPS types in Australia, Brazil, the Netherlands, and Portugal. In Estonia, no cases of MPS IVA were registered [18]. There are no diagnoses of MPS IVB in Kazakhstan to our knowledge.

The most common type in Europe is Sanfilippo’s syndrome (MPS III), comprising 48%, 38%, 44%, and 20% of all MPS types in Poland, Sweden, Germany, and Czech Republic, respectively. In the Republic of Kazakhstan, MPS III is rare, with a birth prevalence of 0.03 per 100,000 live births and presented as the MPS III B type; on the other hand, in European countries, MPS IIIA is the predominant type due to the founder effect. It can be stated that MPS III is the most common MPS type in European populations, whereas MPS II is the most common in Asian countries. According to our results, 49 patients were diagnosed with MPS II in the Republic of Kazakhstan, 90% of them being of Kazakh nationality. However, it is known that MPS III does not show prominent MPS-like phenotypes (coarse face features, macrocephaly, macroglossia, short stature, and dysostosis multiplex) and is characterized by a deterioration in neurological function (neurodegeneration), which manifests at the age of 5–6 years. Considering the aforementioned factors, we could assume that a reason for the rare diagnosis of MPS III in Kazakhstan might be misdiagnosis with other neurological diseases, or even no diagnosis. The rarest type of MPS in Kazakhstan is MPS VII; during the observed period, only one patient was diagnosed with MPS VII in 2023.

In Kazakhstan, before 2015, all MPS patients were diagnosed based on clinical and phenotypical signs, confirmed via enzyme activity assay. Therefore, cases with enzyme pseudodeficiency were excluded from the diagnosed group, since enzyme pseudodeficiency does not typically present disease symptoms.

One of the limitations of our study is that only children with clinical, MPS-like phenotypes underwent enzyme activity assay examination and molecular genetic testing. Some mild forms of MPS are known to not develop typical MPS-like phenotypes. Thus, we assume that the real number and real birth prevalence of MPS are higher in Kazakhstan.

Geographically, the Republic of Kazakhstan is located in the center of Eurasia, mainly in Central Asia, with a territory of 2 million 724.9 thousand km^2^ (nineth largest in the world), inhabited by a population of 20,686,966 people and represented by more than 140 nations and ethnic groups as of the end of 2024. Among them, the vast majority are Kazakhs comprising 70.9% of the population, followed by Russians (14.9%), Uzbeks (3.3%), Ukrainians (1.9%), Uighurs (1.5%), Germans (1.12%), Tatars (1.11%), Turkish (0.45%), and other ethnic groups (4.82%) [28].

According to Kazakh traditions, each Kazakh is obliged to know their ancestors up to the seventh generation for awareness of their genealogy and pedigree. It is forbidden for Kazakhs to marry those who are their relative up to the seventh generation, and thus closely related marriages are practically non-existent in the Kazakh population [30]. However, among other nationalities, such as Uzbeks, Turks, and Tadjiks, consanguineous marriages are common. In our study, we registered three families with consanguineous marriage, who were of Tadjik and Turkish nationalities.

Several recent publications have reported on new MPS-like diseases in 13 patients from Yakutia (Sakha Republic in Russian Federation), 2 siblings from consanguineous marriage in Turkey, and 1 patient each in Poland and Israel. All patients had similar MPS phenotypes and clinical signs, such as coarse facial features, short stature, short neck, respiratory impairment (obstruction of airways, recurrent pneumonia, etc.), skeletal deformities, joint stiffness, intellectual disabilities, and hepatosplenomegaly. The disease is called the “MPS plus” syndrome (OMIM #610034) [29,30,31,32]. Historically, both Yakuts and Turkish nationalities are known to belong to the Turkic ethnic group, and Kazakh people also have Turkic roots. The Kazakh, Yakut, and Turkish languages belong to the Turkic language family and, so, there are many common national customs and traditions. Considering the probable genetic similarity, we can assume that the MPS plus syndrome could be diagnosed in the Kazakh population. The aforementioned findings require detailed examinations of patients with MPS-like phenotypes, but with the absence of laboratorial (lysosomal enzyme diagnosis) confirmation of known MPS types [31,32,33,34].

## 5. Conclusions

Our epidemiological study on MPS birth prevalence in Kazakhstan is the first study focused on Central Asia. Consequently, our data can be useful and important in understanding the estimated prevalence of different MPS types in other Central Asian countries, such as Uzbekistan, Kyrgyzstan, Turkmenistan, and Tajikistan.

In conclusion, the most common type of MPS in the Republic of Kazakhstan is MPS II (Hunter syndrome), of which most patients were of Kazakh nationality, followed by MPS I and VI, while MPS IVA, IIIB, and VII showed the lowest birth prevalence.

## Figures and Tables

**Table 1:** Global birth prevalence of MPS per 100,000 live births.

Country	MPS I	MPS II	MPS III (All Subtypes)	MPS IV (A, B)	MPS VI	MPS VII	MPS IX	MPSX	All Types
Poland [4]	0.22	0.46	0.86	0.14	0.0132	-	-	-	1.81
Netherlands [5]	1.19	0.67	1.89	0.36	0.15	0.24	-	-	4.5
Germany [22]	0.69	0.64	1.57	0.38	0.23	-	-	-	3.51
Australia [10]	1.14	0.74	1.51	0.59	0.43	0.047	-	-	4.46
Norway [6]	1.85	0.13	0.27	0.76	0.07	-	-	-	3.08
Denmark [6]	0.54	0.27	0.43	0.48	0.05	-	-	-	1.77
Sweden [6]	0.67	0.27	0.67	0.07	0.07	-	-	-	1.75
Czech Republic [23]	0.72	0.43	0.91	0.73	0.05	0.02	-	-	3.72
Estonia [18]	-	2.16	1.62	-	0.27	-	-	-	4.05
Japan [24]	0.23	0.84	0.26	0.15	0.03	0.02	-	-	1.53
South Korea [9]	0.21	0.74	0.25	0.13	0.019	-	-	-	1.35
Taiwan [14]	0.11	1.07	0.39	0.33	0.14	-	-	-	2.04
Brazil [19]	0.29	0.48	0.06	0.07	0.35	0.02	-	-	1.57
The United States [25]	0.26	0.26	0.26	0.14	0.04	0.027	-	-	0.98
Saudi Arabi [7]	3.62	-	1.8	3.62	7.85	-	-	-	16.9
Portugal [17]	1.33	1.09	0.84	0.6	0.42	-	-	-	4.8
Mexico [26]	0.19	0.15	0.17	1.1	0.17	0.23	-	-	2.23
Switzerland [24]	0.19	0.46	0.38	0.38	0.11	0.038	-	-	1.56

**Table 2:** The birth prevalence of MPS I, II, IIIB, IVA, and VI in the Republic of Kazakhstan per 100,000 live births (1984–2023).

Type of MPS	Number of Patients	Frequency per Total Quantity of Live Births	Frequency per 10^5^ Live Births
MPS I	22	1 per 621,216	0.16
MPS II	49	1 per 278,913	0.36
MPS III B	4	1 per 3,416,688	0.03
MPS IV A	12	1 per 1,138,896	0.09
MPS VI	17	1 per 803,926	0.12
MPS VII	1	1 per 13,666,750	0.007
All types of MPS	105	1 per 130,160	0.77

**Table 3:** The data of patients with MPS I, II, IIIB, IVA, VI, and VII in the Republic of Kazakhstan.

MPS Types	Number of Severe Cases	Total Number of Patients	Median Age at the Time of Diagnosis (Ranged)	Ethnic Group	Gene	The Most Common Mutations	Mortality
MPS I	11 out of 22 (Hurler syndrome)	22	4 (1–9 years)	Turkish—4.5% Tatar—4.5% Tadjik—9% Russian—18% Kazakh—64%	*IDUA*	c.1709A>T p.Asp570Val c.208C>T p.Gln70Ter	2 cases (respiratory infection, pneumonia)
MPS II	33 out of 49 (neuronopathic form)	49	5.5 (6 months–26 years)	Kyrgyz—2% Russian—8% Kazakh—90%	*IDS*	c.253G>A p.Ala85Thr, hemizygous complex rearrangement between intron 3 and intron 7 of IDS and pseudogene IDS-2, hemizygous deletion including 5 UTR of IDS gene	5 cases (respiratory infection, pneumonia) 1 case (heart failure—dilated cardiomyopathy)
MPS III B	4	4	6.25 (3–12 years)	Russian—50% Kazakh—50%	*NAGLU*	c.454C>T p.Arg152Ter c.1694G>A p.Arg565Gln	1 case due to severe progressive disease course
MPS IVA	1 out of 12 (atlantoaxial instability)	12	8.3 (1–34 years)	Kazakh—92% Turkish—8%	*GALNS*	c.463G>A p.Gly155Arg c.1462G>A p.Val488Met	1 case (severe atlantoaxial instability)
MPS VI	6 out of 17 (severe narrowed craniovertebral junction)	17	4.5 (1–19 years)	Turkish—6.25% Uyghur—6.25% Russian—6.25% Uzbek—12.5% Kazakh—8.75%	*ARSB*	c.275C>A p.Thr92Lys	2 cases (respiratory infection, pneumonia) 1 case (car accident)
MPS VII		1	6 years	Russian—100%	*GUSB*	c.511 G>A p.Ala171Thr	

## Data Availability

Local Scientific center of Pediatrics and Pediatric surgery MPS patient registry.

## Abbreviations

The following abbreviations are used in this manuscript:

MPS	Mucopolysaccharidosis
GAG	Glycosaminoglycans
LSD	Lysosomal storage disease
DBSs	Dried blood spot cards
ERT	Enzyme replacement therapy
